# Prevalence of stress and associated factors among students in Ethiopia: a systematic review and meta-analysis

**DOI:** 10.3389/fpubh.2025.1518851

**Published:** 2025-02-17

**Authors:** Anmut Endalkachew Bezie, Giziew Abere, Girum Tareke Zewude, Belay Desye, Chala Daba, Eyob Tilahun Abeje, Awoke Keleb

**Affiliations:** ^1^Department of Occupational Health and Safety, College of Medicine and Health Sciences, Wollo University, Dessie, Ethiopia; ^2^Department of Environmental and Occupational Health and Safety, Institute of Public Health, College of Medicine and Health Sciences, University of Gondar, Gondar, Ethiopia; ^3^Department of Psychology, Wollo University, Dessie, Ethiopia; ^4^Department of Environmental Health, College of Medicine and Health Science, Wollo University, Dessie, Ethiopia; ^5^Department of Epidemiology and Biostatistics, School of Public Health, College of Medicine and Health Science Wollo University, Dessie, Ethiopia

**Keywords:** high school students, college students, university students, stress in Ethiopia, systematic review, meta-analysis

## Abstract

**Introduction:**

Stress is an increasing public health issue among the student population. This stress affects their academic performance, mental health, and overall well-being. As a result, we conducted a systematic review and meta-analysis to determine the pooled prevalence and associated factors of stress among students in Ethiopia.

**Methods:**

An extensive search of electronic databases such as PubMed, Google Scholar, Semantic Scholar, HINARI, and Science Direct, cross-referencing, and Google manual search was conducted to identify cross-sectional studies published from 1998 to 2024. The preferred PRISMA guideline was used to report items for this systematic review and meta-analysis. To extract data, Microsoft Excel 16 and to analyze STATA 17 software were used. The JBI quality assessment tool was used for the included studies with medium to high-quality scores. To estimate the pooled prevalence of stress and its associated factors, a random effects model was used. The funnel plot and Egger’s regression test were used to assess publication bias, and I^2^ test statistics were used to determine heterogeneity of the included studies. The protocol for this review has been registered with PROSPERO (ID: CRD42024578490).

**Results:**

A total of 23 studies with 8,946 study participants met the inclusion criteria. The pooled prevalence of stress among students in Ethiopia was 37.64% (95% CI: 29.61–45.66; I^2^ = 98.8%). Female gender (OR = 1.82, 95% CI: 1.57–2.12), rural resident (OR = 1.51, 95% CI: 1.22–1.87), living outside university dormitory (OR = 2.02, 95% CI: 1.34–3.05), the habit of alcohol consumption (OR = 1.46, 95% CI: 1.12–1.91), being a cigarette smoker (OR = 2.36, 95% CI: 1.49–3.74), being a khat chewer (OR = 1.35, 95% CI: 1.02–1.80), working in an unfavorable environment (OR = 1.80, 95% CI: 1.20–2.71), and having poor social support (OR = 1.93, 95% CI: 1.39–2.68), were significantly associated with an increased risk of stress.

**Conclusion:**

The findings of this systematic review and meta-analysis indicated a high prevalence of stress among students. The study identified female gender, being a rural residence, living outside a university dormitory, khat chewing, having the habit of alcohol consumption, working in an unfavorable environment, poor social support, and cigarette smoking as significant risk factors for stress. To develop coping skills and resilience, integrating mental health education into the curriculum could help students to prevent stress. Furthermore, strategies such as academic support programs, substance use reduction programs, counseling services, and stress management workshops could be beneficial.

**Systematic review registration:**

https://www.crd.york.ac.uk/prospero/display_record.php?ID=CRD42024578490, CRD42024578490.

## Introduction

Stress is the detrimental physical, social, or psychological reaction that arises when job demands are not aligned with an individual’s capabilities, resources, or requirements (1). It is a current health problem with alarming growth in the workplace and education (2, 3). The World Health Organization highlights that stress is a serious health problem in the category of mental health, which is a significant cause of overall disease burden globally (4). Students feel pressure when they face academic demands that exceed their perceived ability to cope due to heavy workloads, high expectations from parents or teachers, competitive environments, and the challenges of balancing studies with other responsibilities (5).

Evidence from the Health and Safety Executive (HSE) in the United Kingdom suggested that stress is the second most common cause of work-related illness, 20% of workers feel very or extremely stressed at work, and 6.5 million sick days are being taken off (6). Stress causes 40% of job turnover in the United States, and nearly 1 million people miss work every day. Companies lose more than $300 billion each year owing to accidents, absenteeism, lost productivity, and costs associated to healthcare, legal challenges, insurance, and worker’s compensation, all linked to stress (7). Moreover, according to the European Working Conditions survey in 2005 and 2007, 20% of the employees in the first 15 member states of the European Union were stressed (7), and around 40 million persons in the European Union were affected by work-related stress (8).

This trend is mirrored in educational settings, where stress affects both students and faculty. Stress mostly affects students, who are the most productive parts of societies and represent future builders in any country (4, 9). For students to engage effectively in their education, they need to be in good health and mental stability. The International Labor Organization (ILO) also emphasizes that stress-related conditions, including those encountered in academic environments, are a major concern, with long-term effects on mental health such as anxiety, depression, and burnout, which can begin in academic settings and spread to professional life productivity (8). Numerous prior research studies have found that stress impacts students’ learning capacity, academic performance, educational and career success, sleep quality and quantity, substance use and its consequences (10), and overall well-being (10, 11). For example, the United States National College Health Assessment study (2015) found that college students experience significant levels of stress, which affects their mental health and exposes them to stress-related suicidal thoughts (12). Research in Australia, China, and Germany showed that stress has an impact on secondary and postsecondary students’ academic achievement (13–15). Moreover, studies in China and Japan revealed that academic stress negatively impacts intrinsic academic motivation and self-development (16–18).

In Ethiopia, the rapid expansion of education and the increasing demands on students have increased concerns about stress prevalence and associated factors across different educational levels. High school and college or university students encounter a variety of continuous normative stressors, which are characterized as typical daily inconveniences and ongoing academic demands (10). Previous research in Ethiopia found that students suffer specific pressures (4, 19, 20). The high societal value placed on education as a road to social mobility and economic stability frequently leads in increased academic expectations from family and community members. This cultural emphasis adds pressure on students to excel academically. From a socioeconomic perspective, many Ethiopian students confront significant financial obstacles, including scarce resources that may result in poor living conditions and limited access to educational resources (21). The nation’s overall economic volatility exacerbates these financial difficulties and also heightens concerns about future employment opportunities. Additionally, there is inequality in educational infrastructure between urban and rural areas (22), high school, college, and university. This means students from less developed regions may experience greater stress because of inadequate educational facilities and social support systems. These cultural and socioeconomic factors create a unique and multifaceted stress landscape for Ethiopian students, necessitating tailored interventions and support systems.

As students’ progress through the educational stage, they encounter new academic constraints such as a heavy course load, intensive study sessions, managing their time effectively, classroom rivalry, financial worries, and familial demands. Moreover, as students move into a new school, they take ownership of their education and encounter a range of challenges, such as acclimating to the culture and environment of the college or university, strained inter and intrapersonal relationships, a demanding curriculum, and little institutional support. Because of this, they are typically very sensitive to stress. This stress can negatively impact their future careers by causing them to perform poorly, drop out of school, become absentees, and be less productive (10). University students, particularly in demanding, highly preferred programs like medicine, architecture, and software engineering, experience significant stress, which often persists through practical training that impacts their mental health, and academic performance (23–25).

College or university life can also be stressful due to a variety of factors, including biological factors like age and gender, particularly being female (26), marital status, academic performance or pressure from things like exams and workload, lack of free time, competition, and worries about not living up to parents’ expectations (27), and financial burden (28). Personal traits, grade level, type of study, living conditions, work environment, chronic illness, and undergoing significant life changes can exacerbate stress (29). Infrequent exercise, alcohol consumption, smoking, khat chewing, sleep disturbances, and poor dietary habits are linked to elevated stress (30, 31). Stress affects students through various physiological, psychological, and behavioral mechanisms. Stress physiologically causes the release of chemicals such as cortisol and adrenaline, which, if encountered repeatedly, can damage cognitive abilities and weaken the immune system (23). Stress affects concentration and attention psychologically, making it harder to focus on schoolwork and remember knowledge. Behaviorally, stress often results in avoidance of responsibilities, unhealthy coping strategies, and bad lifestyle choices (32).

Researchers have found that excessive stress produces serious side effects, including the desire to cheat on exams, reduced academic performance, trouble resolving interpersonal conflicts, diminished focus, a higher chance of making mistakes, neglect, sleep issues, and low self-esteem (10). Therefore, policy-level interventions specific to Ethiopia are highly required. These may include implementing institutional support systems, addressing gender-specific stresses, ensuring equitable access to resources for rural or low-income communities, and managing stress through time management, mindfulness, and good lifestyle choices.

Despite the global recognition of student stress as a serious public health concern, there is still a paucity of comprehensive research on its prevalence and associated factors in Ethiopia. In addition, prior literatures have some methodological gaps. Some studies relied on small, non-representative samples, mostly limited on certain institutions or region. This restricts the relevance of result to the diverse experiences of students across the country. Furthermore, cross-sectional study designs dominate the existing literature that provides only a snapshot of stress levels at a single point in time without considering the dynamic nature of stress. To fill these methodological inadequacies, our systematic review and meta-analysis followed strict inclusion criteria to ensure a complete and representative sample of research from diverse educational levels and regions in Ethiopia. Therefore, the aim of this systematic review and meta-analysis was to determine the pooled prevalence and associated factors of stress among students in Ethiopia. The findings of this review will provide evidence-based insights to guide stress-reduction strategies. Moreover, it contributes to the academic discourse on student stress and can serve as a baseline for educators, policymakers, and mental health practitioners so as to promote students’ mental health and academic success.

## Methods

### Reporting system and registration

We conducted a systematic review by targeting primary studies on the prevalence of stress and associated factors among students in Ethiopia. This review adhered to the core principles outlined in the Center for Reviews and Dissemination’s (CRD) guidance for healthcare reviews and followed the Preferred Reporting Items for Systematic Reviews and Meta-Analyses (PRISMA) guideline. The review protocol was registered with the International Prospective Register of Systematic Reviews (Record ID: CRD42024578490).

### Data sources, study period, searching strategies, and study selection

We conducted a thorough literature search of cross-sectional studies across multiple electronic databases, including PubMed, Google Scholar, HINARI, Semantic Scholar, and Science Direct. We included studies published from the inception of these databases in 1998 up to August 30, 2024, by seven authors independently (AEB, AK, GA, GTZ, BD, CD, and EAT). Studies from previous systematic reviews were reassessed and incorporated. To ensure a comprehensive search, we also meticulously reviewed the references in selected studies to identify any related studies that may have been missed. The MeSH and search filters were included in the search strategies developed via the PMC Advanced Search Builder with the following keywords and Boolean operators: ((((((((((((((((((((((((((prevalence[Title/Abstract]) OR (magnitude[Title/Abstract])) OR (proportion[Title/Abstract])) OR (epidemiology[Title/Abstract])) AND (stress*[Title/Abstract])) OR (“psychological stress”[Title/Abstract])) OR (“perceived stress”[Title/Abstract])) OR (“emotional stress”[Title/Abstract])) OR (“mental stress”[Title/Abstract])) OR (“academic stress”[Title/Abstract])) OR (“stress disorders”[Title/Abstract])) AND (student*[Title/Abstract])) OR (“high school students”[Title/Abstract])) OR (“secondary school students”[Title/Abstract])) OR (“college students”[Title/Abstract])) OR (“university students”[Title/Abstract])) OR (“undergraduate students”[Title/Abstract])) OR (“nursing students”[Title/Abstract])) OR (“postgraduate students”[Title/Abstract])) OR (“adolescent”[Title/Abstract])) OR (“young adults”[Title/Abstract])) AND (“associated factors”[Title/Abstract])) OR (“risk factors”[Title/Abstract])) OR (“predictors”[Title/Abstract])) OR (“determinants”[Title/Abstract])) OR (correlates[Title/Abstract])) AND (Ethiopia[Title/Abstract]; Supplementary file1). Beyond the primary keywords, we employed synonyms, abbreviated symbols, and additional free-text keywords to enhance the search. Only full-text articles published in English were included in the review. The reference lists of selected articles were manually examined, and the “similar articles” feature of the databases was utilized to identify additional relevant studies. All included and excluded studies were screened using EndNote 20 and the Rayyan automation tool. The screening process began with an independent review of titles and abstracts, followed by a full-text screening of the selected studies by seven authors. Any disagreements were resolved through consensus. The selection process was meticulously documented to enable the completion of a PRISMA 2020 flow diagram.

### Inclusion and exclusion criteria

Cross-sectional studies examining stress prevalence and associated factors among students in Ethiopia were included in this systematic review and meta-analysis. We included studies without restrictions on the study period, sample size, study setting in Ethiopia and published research. Studies that used validated and reliable stress measurement tools were included to ensure the reliability and comparability of the data. Exclusion criteria were applied to studies that did not report on stress prevalence or its associated factors within the specified population, unrelated research, and studies where the full text was not available or retrieved. We also excluded duplicate data sources and studies with poor methodological quality. Qualitative studies, studies other than prevalence or proportion studies, editorial letters, and non-research articles were also excluded.

### Outcome assessment

Estimating the pooled stress prevalence among students in all educational sectors in Ethiopia was the primary objective, and finding factors associated with stress in this population expressed as odds ratio was the secondary outcome measurement.

### Data extraction and quality assessment

After all articles were exported into EndNote 20 and the Rayyan automation tool, duplicate entries were removed. The remaining data were extracted using a standardized form, which was initially piloted on two included studies. This form captured study characteristics and outcomes and was implemented in Microsoft Excel 2016. Seven authors (AEB, AK, GA, GTZ, BD, CD, and ETA) were responsible for extracting data from cross-sectional studies, recording details such as the authorship, publication year, school type, study design, sample size, timing of outcome measurement, stress prevalence, and its associated factors among students. Following the screening of relevant articles for eligibility by the seven reviewers, the quality of each study was assessed using the Joanna Briggs Institute (JBI) critical appraisal checklist. Each reviewer independently evaluated the risk of bias for the studies, with the results expressed on a 100% scale. Articles with a quality score above 50% were included in the subsequent qualitative and quantitative analyses. To resolve any differences that arose during the quality assessment, the mean score from all reviewers was calculated.

### Publication bias assessment

To assess publication bias of the included studies, we employed Begg’s funnel plots and Egger’s test. First, we visually examined the symmetry of the funnel plots. Second, we quantitatively assessed the likelihood of publication bias using Egger’s regression test.

### Data analysis and synthesis

All analyses were conducted using Stata version 17. To estimate the pooled prevalence of stress and its associated factors, we employed random effects models. The I^2^ statistic was used to examine heterogeneity among the incorporated studies, with I^2^ values below 50% indicating homogeneity and values of 50% or higher indicating significant heterogeneity. We considered a 95% confidence interval (CI) and a *p*-value of less than 0.05 as statistically significant for associations, absence of publication bias, and heterogeneity. For the summary measure, we utilized a random effects model, assuming that the included studies represent a random sample of all possible results. We used the DerSimonian-Laird estimator with the Knapp and Hartung adjustment for standard errors.

Subgroup analyses were conducted to investigate potential sources of heterogeneity, focusing on factors such as study year (before and after COVID-19), and school type (high school, college, and university). Additionally, a series of sensitivity analyses were performed to examine the validity and robustness of the summary measures. First, we applied the random-effects model while excluding studies based on their JBI scores, specifically removing those with high and intermediate risk of bias. Second, an outlier analysis was conducted to identify studies with extreme effect sizes that could potentially skew the overall conclusions. Outliers were defined as those with a studentized deleted residual greater than 1.96. Third, a sensitivity analysis such as a leave-one-out was performed by gradually eliminating each study to ensure that the overall findings were not unduly influenced by any single study. All analyses were done using the “metan” package in the Stata software version 17.^1^

## Results

### Searching process

**Figure 1:**
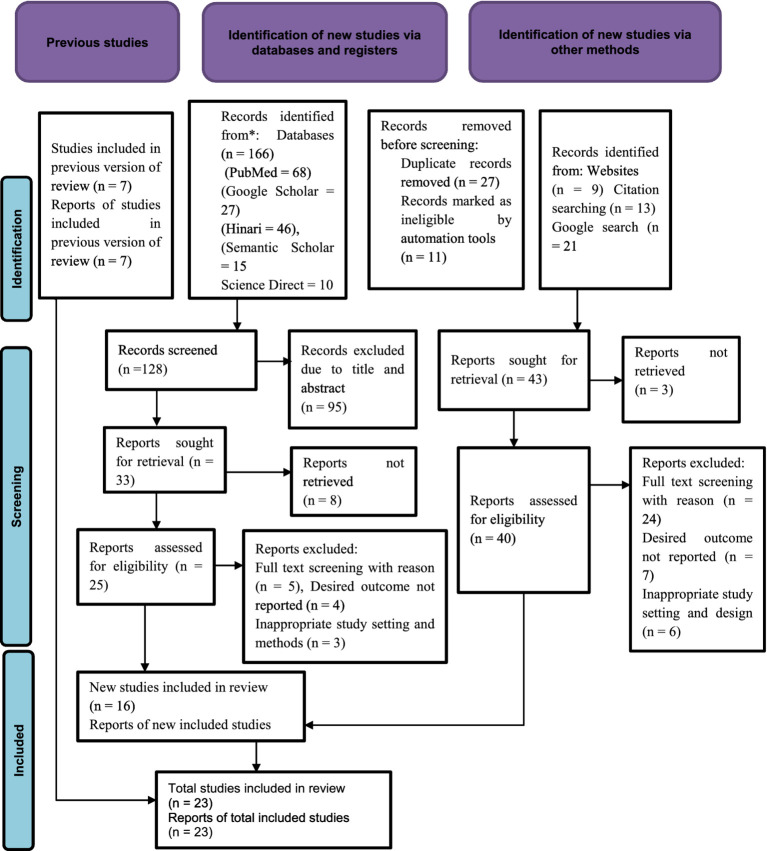
PRISMA flow diagram for the systematic review and meta-analysis of stress prevalence and associated factors among students in Ethiopia.

A total of 216 articles were identified using electronic databases and manual searching. After removing 27 duplicate records, 11 records were marked as ineligible through automation tool. Based on their titles and abstracts 95 articles were excluded. In addition, 49 articles were excluded based on the exclusion criteria. Finally, a total of 23 articles were included in this review. The PRISMA flow diagram was used to summarize the selection process (Figure 1).

### Characteristics of the included studies

In this review, the publication year, study setting, study design, sample size, and prevalence of stress are summarized in Table 1. By design, all included studies were cross-sectional. This study included a total of 8, 946 participants (4, 19, 20, 23, 33–51). The included articles were published between 2015 and 2024 with sample sizes ranged from 153 to 810. In this review, a study conducted in Debre Birhan Governmental and Nongovernmental Health Science Colleges students found the lowest (4.1%) (34), and a study conducted among Debre Birhan University students was the highest prevalence of stress (63.7%) (20). Of the research, two were done with high school students (35, 49); three were done with college students (33, 34, 45); and the remaining 18 studies were done with university students (4, 19, 20, 23, 36–44, 46–48, 50, 51). The incorporated studies were categorized as having a low probability of bias (quality score 5 to 8). Table 1 presents a description of the incorporated studies.

**Table 1:** Shows the prevalence of stress and its characteristics among students in Ethiopia.

No.	Author (publication year)	study setting	Study year	Study design	School type	Sample size	Response rate	Prevalence (95% CI)	Quality status
1	Tadesse et al., 2021 (33)	Amhara regional	2020	cross sectional	college	408	96.7	48.5	Low risk
2	Abebe et al., 2018 (34)	Debre Birhan	2016	cross sectional	College	416	98.6	4.1	Low risk
3	Worku et al.,2020 (4)	Arsi	2019	cross-sectional	University	384	100	63.5	Low risk
4	Nakie et al., 2022 (35)	Northwest Ethiopia	2021	cross-sectional	high school	810	96.1	52.2 (49.1, 56)	Low risk
5	Asfaw et al., 2021 (36)	Haramaya	2019	cross-sectional	university	523	94	44 (40.2, 48.4)	Low risk
6	Melaku et l 2021 (19)	Arsi	2019	cross-sectional	University	260	98.1	40.4	Low risk
7	Damota et al., 2019 (37)	Madda Walabu	2015	cross sectional	university	384	100	50.3	Low risk
8	Suleyiman et al., 2018 (38)	Ambo	2015/2016	cross-sectional	University	343	85.1	24.5	Low risk
9	Damota et al., 2018 (39)	Addis Ababa	2013	cross sectional	University	259	100	24	Low risk
10	Simegn et al., 2022 (40)	Ethiopia	2021	cross sectional	University	426	100	18.3	Low risk
11	Simegn et al., 2021 (41)	Ethiopia	2020	cross sectional	University	423	100	28.6 (24.6, 32.9)	Low risk
12	Kasa et al., 2017 (42)	Bahir Dar	2016	cross-sectional	University	254	100	37.4	Low risk
13	Madebo et al., 2016 (20)	Debre-Birehane	2013	cross sectional	University	273	98	63.7	Low risk
14	Awoke et al., 2021 (43)	Jimma	2020	cross sectional	University	337	100	35.9	Low risk
15	Ezo et al., 2024 (44)	Wachemo	2024	cross-sectional	University	421	100	58.4 (53.6, 62.8)	Low risk
16	Sahile et al., 2020 (45)	Addis Ababa	2020	cross sectional	College	153	22.5	11.1 (6.6, 17.2)	Low risk
17	Melaku et al., 2015 (23)	Jimma	2015	cross sectional	University	317	96.4	52.4	Low risk
18	Henok Shiferaw et al. 2015 (46)	Jimma	2013/2014	cross-sectional	University	193	100	47.7	Low risk
19	Zegeye et al., 2018 (47)	Jimma	2016	cross sectional	University	346	96.1	46.2(40.75, 51.3)	Low risk
20	Aylie et al., 2020 (48)	Bench-Sheko Zone	2020	cross-sectional	University	314	97.5	32.5 (27.1, 38.2)	Low risk
21	Abera et al., 2023 (49)	Sawla town, Gofa zone	2021	cross-sectional	High-school	654	98.6	22.6 (19.4, 25.9)	Low risk
22	Mekonen et al., 2021 (50)	Gondar	2020	cross-sectional	University	338	96.6	22.2 (17.8, 26.6)	Low risk
23	Assefa et al., 2021 (51)	Wolkite	2020/2021	cross-sectional	university	710	95.6	38.2	Low risk

### Assessment methods of stress

Studies using various stress screening techniques were included in this systematic review. Standardized questionnaires were widely used, but there was no clinical confirmation or longitudinal research in any of the investigations, which raised doubts about the precision of stress assessment. Most of the study used the depression anxiety and stress scale to measure depression anxiety and stress; some of the study used the perceived stress scale, and others used the general health survey. Some studies used a combination of qualitative and quantitative assessment methods. To handle the differences in prevalence’s used by various stress scales, we performed sensitivity analyses to assess the impact of these variations. Additionally, for studies using less common or newer stress measurement tools, we ensured that each tool had been validated in the context of the specific population being studied. In cases where the validity or reliability of the tool was unclear, we excluded these studies from the analysis. This approach ensured that despite the use of different stress measurement tools, we were able to provide a robust and comparable analysis of stress among students in Ethiopia. Table 2 provides a summary of the assessment methods used in the original studies.

**Table 2:** Sampling technique, data collection tool, and data collection methods used in the original studies to assess the levels of stress in Ethiopia.

No.	Author (publication year)	Sampling technique used to select study participants	Data collection tool used to measure stress	Methods of data collection
1	Tadesse et al., 2021 (33)	systematic random	DASS questioner	phone call and personal interviews
2	Abebe et al., 2018 (34)	stratified and systematic random	DASS	interviewing with using semi structured questionnaire
3	Worku et al.,2020 (4)	stratified and simple random	PSS-14	Self-administered
4	Nakie et al., 2022 (35)	stratified multi-stage and simple random	DASS-21 questionnaire	Self-administered
5	Asfaw et al., 2021 (36)	simple random	DASS	Self-administered
6	Melaku et l 2021 (19)	stratified and systematic random	DASS	Self-administered
7	Damoto et al., 2019 (37)	stratified	DASS	Self-administered
8	Suleyiman et al., 2018 (38)	simple random	Student-life Stress Inventory	unstructured and semi structured interview
9	Damota et al., 2018 (39)	proportionate stratified random	DASS	Self-administered
10	Simegn et al., 2022 (40)	Snowball	PSS (10 items)	Through social media (Telegram groups, Imo, Emails, face book)
11	Simegn et al.,2021 (41)	voluntary participation (absence of random sampling)	DASS	Google forms
12	Kasa et al.,2017 (42)	simple random	PSS (29 items)	Self-administered
13	Madebo et al., 2016 (20)	stratified and simple random for quantitative part and convenient for qualitative part	PSS (pss-14)	Both qualitative and quantitative
14	Awoke et al., 2021 (43)	Both purposive and snowball	PSS (10 items)	Google Forms
15	Ezo et al., 2024 (44)	Proportional allocation to the department and simple random	PSS (29 items)	Self-administered
16	Sahile et al., 2020 (45)	through WhatsApp, Telegram, Messenger, and email	DASS 21	online data collection tool Google forms
17	Melaku et al., 2015 (23)	proportional allocation method followed by simple random	GHQ-12, Medical Students Stress Questionnaire	Self-administered
18	Henok Shiferaw et al. 2015 (46)	proportionate stratified random	Perceived stress scale (10 items)	Self-administered
19	Zegeye et al., 2018 (47)	stratified followed by simple random	(GHQ-12) and Postgraduate Stressor Questionnaire (PSQ-28)	Self-administered
20	Aylie et al., 2020 (48)	systematic random	DASS-questionnaire	interviewed via phone calls
21	Abera et al., 2022 (49)	proportionally allocated based on the total number of students followed by simple random	DASS	face-to-face interviews with open data kit (ODK) tools
22	Mekonen et al., 2021 (50)	proportional allocation followed by simple random	DASS	Self-administered
23	Assefa et al., 2021 (51)	stratified followed by simple random	DASS	Self-administered

DASS, Depression, Anxiety, and Stress Scale; PSS, Perceived Stress Scale; GHQ, General Health Questionnaire.

## Meta-analysis

### Pooled prevalence of stress among students in Ethiopia

**Figure 2:**
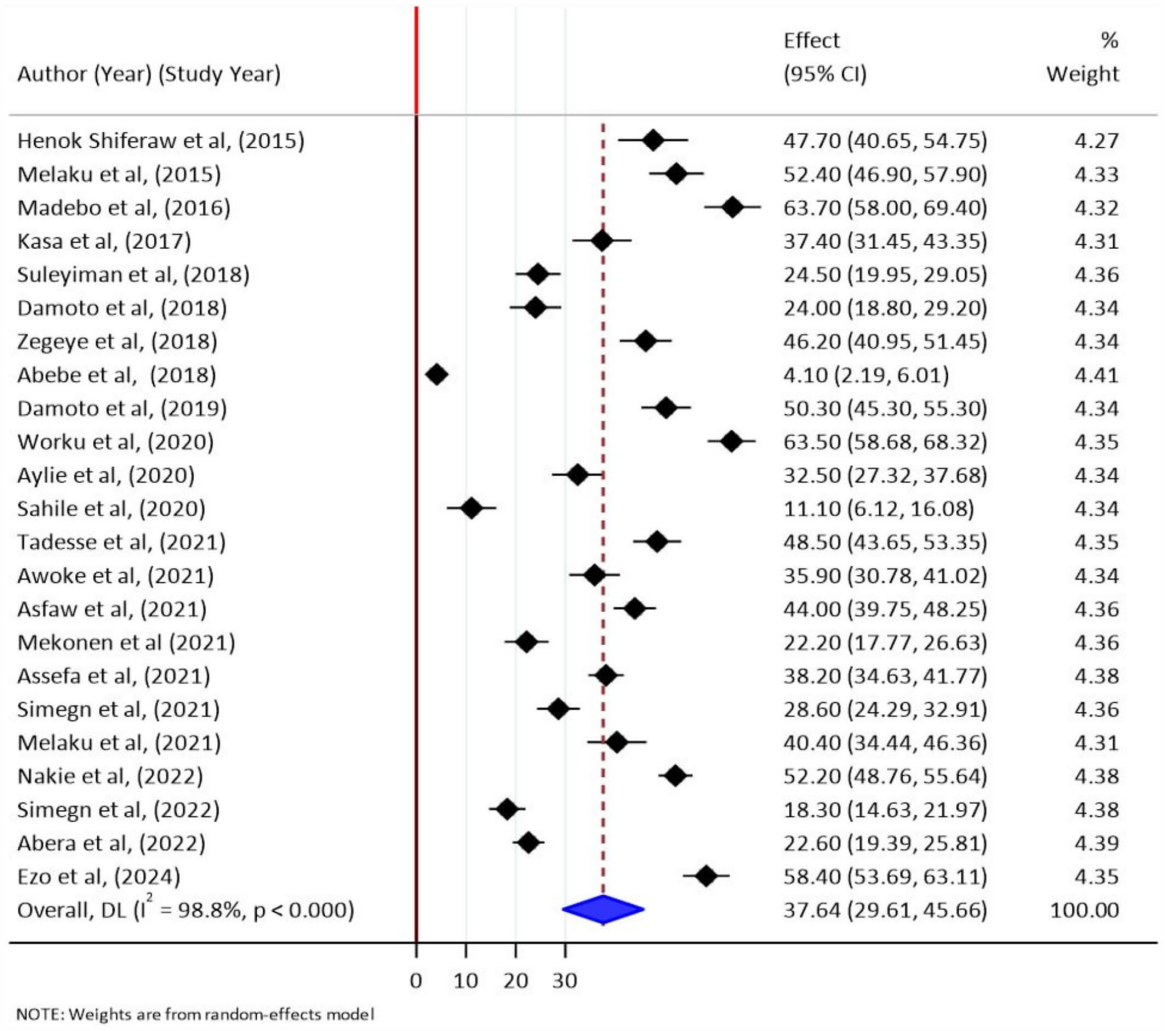
A forest plot shows the prevalence estimates of stress among students in Ethiopia, 2024.

The pooled prevalence estimate of stress was found to be 37.64% (95% CI: 29.61–45.66; I^2^ = 98.8%). In this analysis, the lowest prevalence of stress was found among Debre Birhan college students at 4.10% (95% CI: 2.19–6.01) (34) and the highest prevalence of stress was found among Debre Birhan University health science students at 63.7% (95% CI: 58.68–68.32) (20). A forest plot shows the prevalence estimates of stress among students in Ethiopia (Figure 2).

### Subgroup analysis

**Figure 3:**
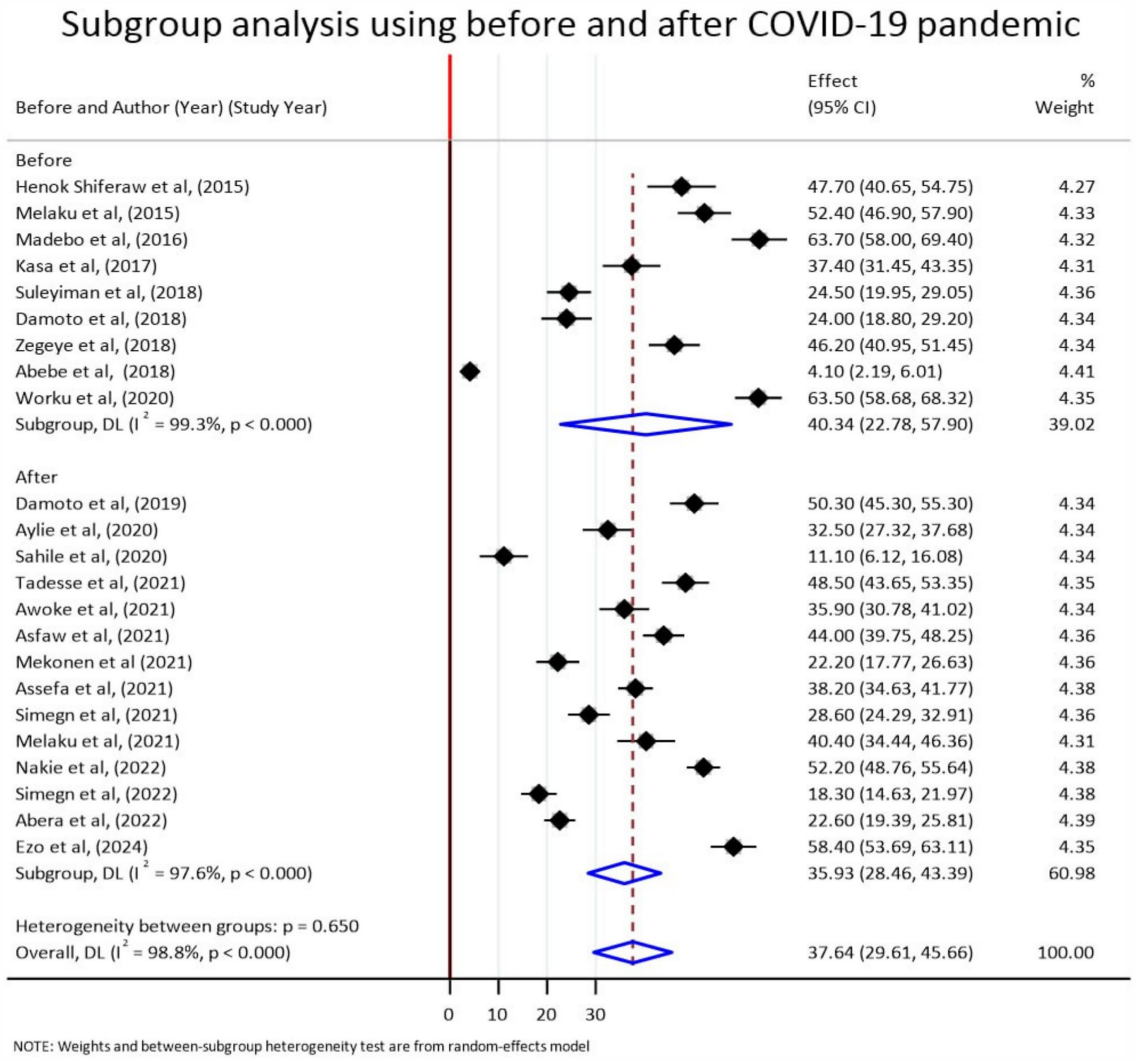
Subgroup analyses by publication years (before and after COVID-19) for the pooled prevalence of stress among students in Ethiopia, 2024.

**Figure 4:**
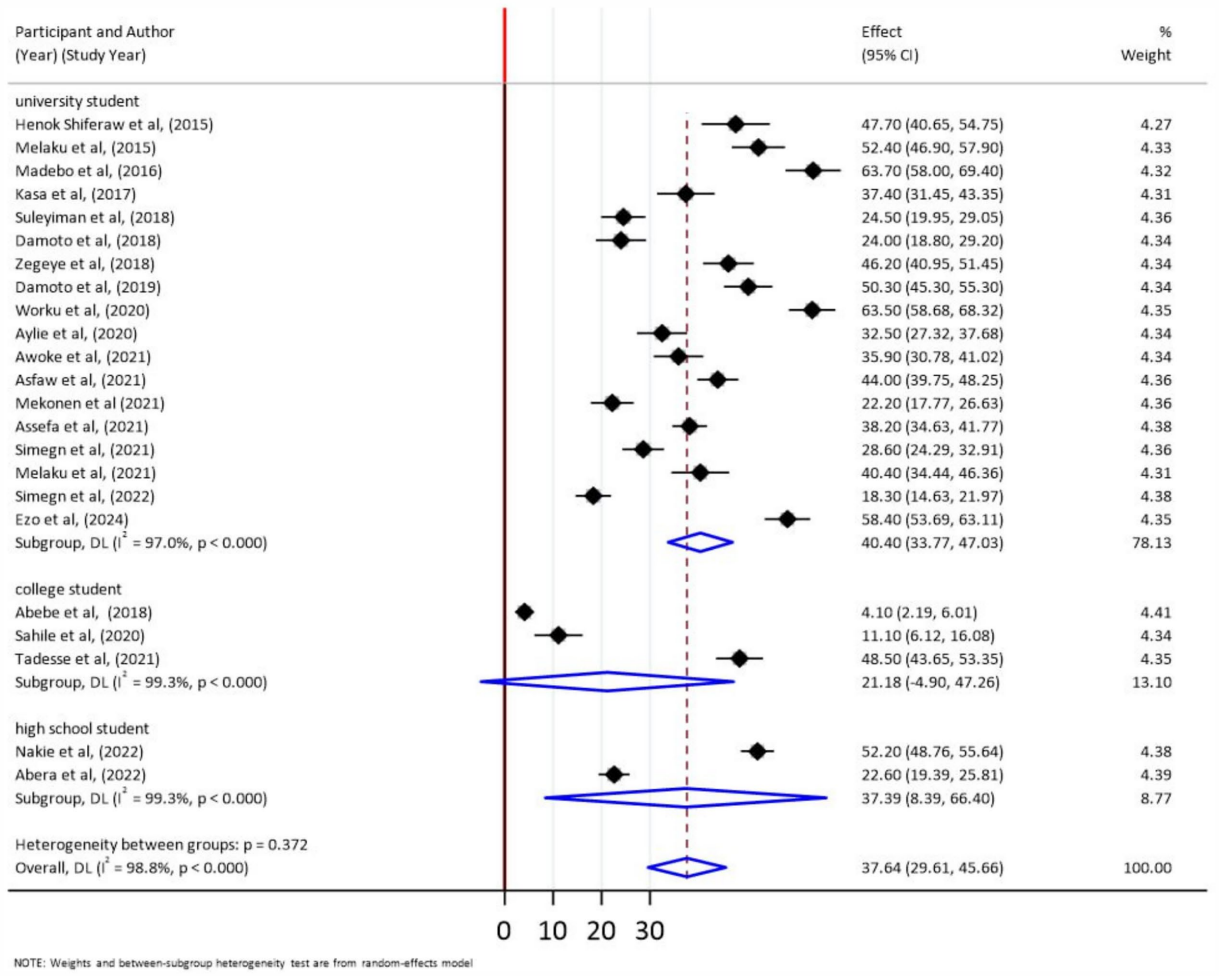
Subgroup analyses by school types for the pooled prevalence of stress among students in Ethiopia, 2024.

According to the subgroup analysis result, the pooled prevalence of stress before and after COVID-19 was 40.34% (95% CI: 22.78–57.9) and 35.93% (95% CI: 28.46–43.39), respectively (Figure 3). A subgroup analysis was also performed with school type (university, college, high school). Accordingly, the pooled prevalence of stress was 40.4% (95% CI: 33.77–47.03), 21.18% (95% CI: −4.90, 47.26), and 37.39% (95% CI: 8.39–66.40), respectively (Figure 4).

### Heterogeneity and publication bias

**Figure 5:**
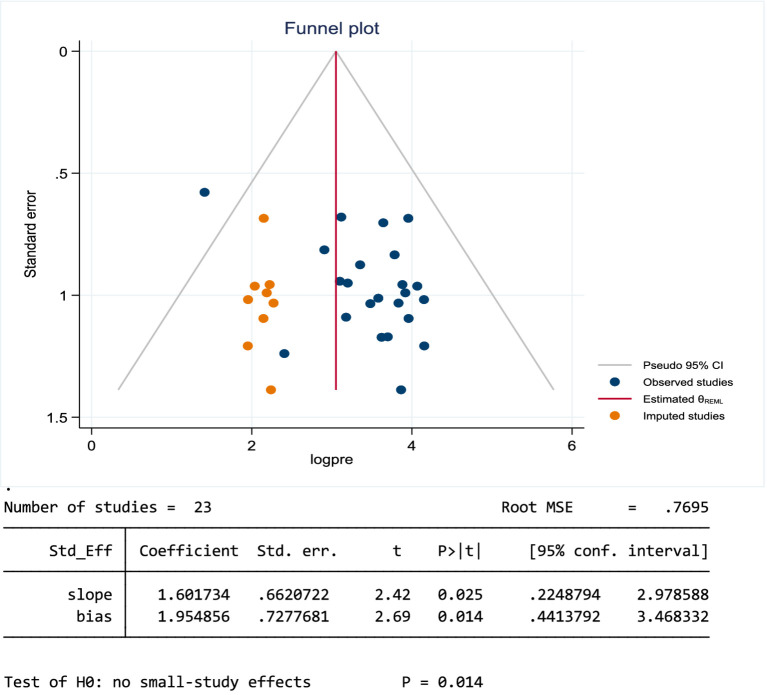
Funnel plot and Egger’s test of studies reporting stress among students in Ethiopia, 2024.

The presence of heterogeneity and publication bias (small study effect) was assessed within the included studies. Publication bias occurs when research with significant results is more likely to be published than those with no significant results. Egger’s test and Begg’s funnel plots were used to evaluate any potential publication bias quantitatively. The included studies had a high degree of heterogeneity (I^2^ = 98.8%, *p* < 0.001). Publication bias was assessed using a funnel plot and Egger’s regression test at a *p* value *<*0.05. The funnel plot showed that the distribution of studies was asymmetrical, whereas Egger’s test was found to be statistically significant (*p* = 0.014) for the estimated prevalence of stress, meaning that there was evidence of publication bias. The trim-and-fill method by Duval and Tweedie was applied to estimate the number of studies potentially missed due to publication bias. The analysis suggested that nine studies were imputed to account for potential publication bias (Figure 5).

**Figure 6:**
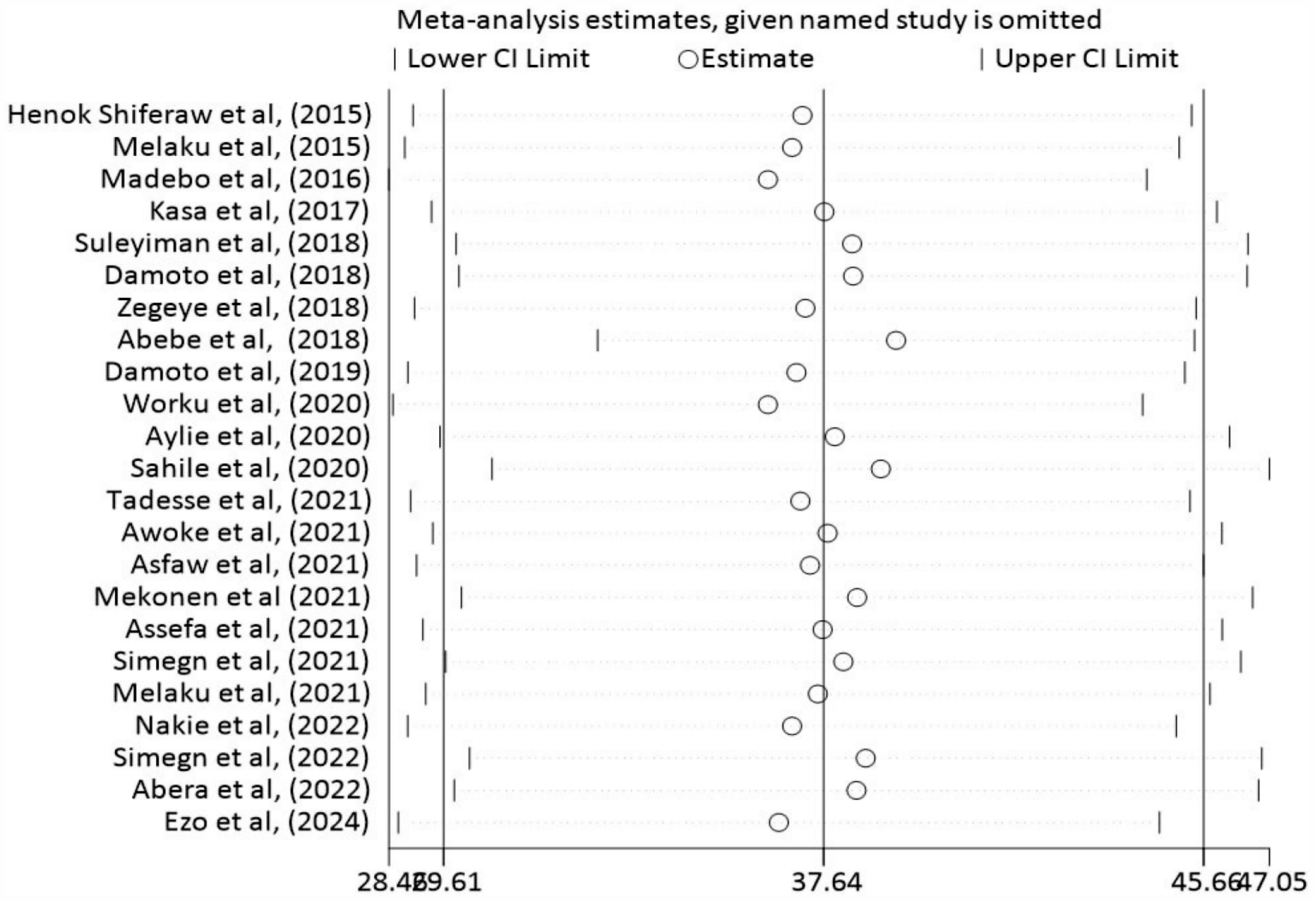
Sensitivity analysis graph to examine the effect of each study on the pooled result of stress, 2024.

A sensitivity analysis was performed to assess the effect of each study on the pooled estimate of Stress. We carefully excluded studies with risk of bias and examined how various measures of size impacted the overall results. The sensitivity analysis revealed that the size remained consistent around (37.64, 95% CI: 29.61, 45.66) and none of the studies seems to be an extreme outlier that shifts the overall estimate significantly. These results highlight the importance of considering the quality and sample size of studies in this meta-analysis and imply robustness of the studies. This supports the robustness of the results (Figure 6).

### Factors associated with stress among students in Ethiopia

This review grouped the associated factors into the following categories: Demographic factors included sex, residency, marital status, and department. Behavioral factors included alcohol use, khat chewing, and cigarette smoking. Academic factors were based on grade levels (9, 10, 11 and 12). Social and health-related factors, including medical illness and social support. Environmental factors such as living off-campus and unfavorable environment. Factors related to stress were found using the combined effect of two or more studies. In this meta-analysis, factors associated with stress were assessed using 23 studies (4, 19, 20, 23, 33–51). The analysis showed that in nine of these studies (19, 34, 36, 40, 41, 44, 47, 49, 50), female students were 1.8 times more likely to be stressed than male counterparts (OR = 1.82, 95% CI: 1.57–2.12). Similarly, the pooled findings of the five studies (35, 40, 43, 49, 50) indicated that students who are in rural residence were 1.5 times more likely to be stressed than their urban counterparts (OR = 1.51, 95% CI: 1.22–1.87). In addition, the pool effect size finding of four studies (19, 23, 36, 47) showed that students who are alcohol drinkers were about 1.5 fold more likely to be affected by stress than nondrinkers (OR = 1.46, 95% CI: 1.12–1.91). Besides, the pooled effect size of the 5 studies (19, 23, 36, 40, 47) showed that khat chawing is the risk factor for stress, and these who chaw khat were nearly 1.4 times more likely to be affected by stress than the none users (OR = 1.35, 95% CI: 1.02–1.80). Moreover, the pooled results of four studies (19, 23, 36, 47) in this meta-analysis revealed students who smoke cigarettes were about 2.4 times more highly affected by stress than these who are not smokers (OR = 2.36, 95% CI: 1.49–3.74). Two studies (4, 34) in this systematic review and meta-analysis highlight that students working in an unfavorable environment were 1.8 fold higher stressed than their counterparts (OR = 1.8, 95% CI:1.20–2.71). Furthermore, in terms of living situation, two studies (19, 36), indicated that students who live off campus were 2 times more likely to develop stress than students who live in university dormitory (OR = 2.02, 95% CI: 1.34–3.05). Finally, two studies (35, 49) in this systematic review and meta-analysis highlight that students with poor social support were 1.9-fold more likely to be stressed than students with strong social support (OR = 1.93, 95% CI: 1.39–2.68; Table 3).

**Table 3:** Factors associated with stress among students in Ethiopia (Pooled OR with 95% CI), 2024.

Factors (not reference)	Number of papers (Reference)	Pooled OR (95% CI)
Sex (female)	9 [19, 33, 35, 39, 40, 43, 46, 48, 49]	1.82 (1.57, 2.12)
Marital status (single)	4 [19, 35, 46, 50]	0.98 (0.77, 1.26)
Department (others)	3 [39, 40, 49]	1.05 (0.68, 1.62)
Residency (rural)	5 [34, 39, 42, 48, 49]	1.51 (1.22, 1.87)
Alcohol use (yes)	4 [19, 22, 35, 46]	1.46 (1.12, 1.91)
Khat chawing (yes)	5 [19, 22, 35, 39, 46]	1.35 (1.02, 1.80)
Cigarette smoking (yes)	4 [19, 22, 35, 46]	2.36 (1.49, 3.74)
Medical illness (yes)	4 [20, 33, 47, 49]	1.36 (0.81, 2.27)
Grade (9) Grade (10) Grade (11)	2 [34, 48]	1.5 (1.02, 2.20)
	2 [34, 48]	1.30 (0.86,1.96)
	2 [34, 48]	1.58 (1.07, 2.33)
Living situation (off campus)	2 [19, 35]	2.02 (1.34, 3.05)
Environment (unfavorable)	2 [4, 33]	1.80 (1.20, 2.71)
Social support (poor)	2 [34, 47]	1.93 (1.39, 2.68)
Social support (moderate)	2 [34, 47]	1.12 (0.93, 1.35)

Only those factors that were assessed by at least two studies were considered for meta-analysis.

## Discussion

This systematic review and meta-analysis was aimed at investigating the pooled prevalence and associated factors of stress among students in Ethiopia. As a result, the pooled prevalence of stress in this systematic review and meta-analysis was 37.6% (95% CI: 29.61–45.66%). The study identified being female, alcohol drinking, cigarette smoking, khat chewing, working in an unfavorable environment, poor social support, being in a rural residence, and living outside the university dormitory as significant factors associated with student stress. The pooled prevalence of stress in this review was almost aligned with the studies carried out among the student population (31%) (52), and medical students in China (34%) (53), Malaysia (33.3%) (54), and college and university students in Ethiopia during COVID-19 (31.43%) (55). This commonality may be due to students encountering common pressures, such as academic restraints like excessive workloads, high expectations from parents and educators, and competitive academic environments. Furthermore, methodological similarities in stress measurement instruments, such as validated questionnaires, may result in comparable prevalence rates across studies.

### Global implications

The findings of this study in Ethiopia have significant implications at the global level. Since this alignment of pooled prevalence of stress indicates that student stress is a widespread issue across diverse geographical and socio-economic contexts, it thereby contributes to the global understanding of student mental health trends. In addition, analyzing individual risk variables provides useful global and cultural insights. For example, female gender and alcohol intake are consistent with findings from research around the world, emphasizing the importance of gender-sensitive approaches and substance use interventions in global student mental health initiatives. In contrast, the unique cultural element of khat chewing emphasizes the significance of adapting interventions to individual groups for increased efficacy. These findings also have significant policy implications, including comprehensive and context-specific treatments to address academic, socioeconomic, lifestyle, and environmental stressors. Furthermore, this study contributes to the global knowledge base by expanding on the limited number of meta-analyses on student stress in low-and middle-income countries.

Alternatively, the pooled prevalence of stress in this review was lower than that of the systematic review and meta-analysis done among nursing professionals in Ethiopia (49.6%) (56), European students’ 62% (57) and cross-sectional study in Brazil (57.5%) (58). The possible reason could be a difference in methodology approaches. The studies were carried out with different populations in different cultural and social contexts that have an effect on managing stress. In contrast, the estimated prevalence of stress in the current review was higher than the studies carried out among nursing (3.7%) (59) and college students in China (23%) (60). This may be due to cultural and socioeconomic status differences, tool differences in assessment of stress, variation in lifestyle factors, academic and institutional support, access to mental health care and counseling services.

### Subgroup analysis by study period

The subgroup meta-analysis of this review indicated that the pooled prevalence of stress before COVID-19 was 40.34% (95% CI: 22.78–57.9) and after COVID-19 was 35.93% (95% CI: 28.46–43.39). Even if this result is counterintuitive, the probable reason for the higher stress levels before COVID-19 was likely due to intense academic pressures and social dynamics (5), while stress during the pandemic decreased due to the flexibility of online classes and reduced commute times to be less stressful than the rigid schedules of in-person schooling. After the pandemic, ongoing adaptations such as many educational institutions implemented changes to accommodate new realities, such as more flexible deadlines and increased mental health support that may help to reduce stress (61).

### Subgroup analysis by school type

According to the result, the pooled prevalence of stress was 40.4% (95% CI: 33.77–47.03), 21.18% (95% CI: −4.90–47.26), and 37.39% (95% CI: 8.39–66.40) among university, college, and high school students, respectively. This indicated that the highest prevalence was among university students (40.4%) and the lower was from college students (21.18%). This could be because university students are under more stress than college and high school students due to more stringent academic requirements, such as more difficult courses, longer reading assignments, and frequent high-stakes exams (62). In addition, the adjustment to university life typically includes the first time they live away from their parents. This change requires them to manage their finances, housing, and daily routines without parental aid, which increases their stress. They are also getting closer to entering a competitive job market, which raises concerns about their future employment, career options, and financial security (63). Moreover, higher expectations from family, society, and students themselves also contribute to a stressful environment because attending university raises demands from all of these groups (63). Furthermore, financial strain related to worries about tuition fees, student loans, and other expenses such as limited availability of scholarships and financial aid in Ethiopia may exacerbate stress.

Furthermore, the higher prevalence of stress among high school adolescents than college students could be attributed to high school students’ high academic pressure from important national exams such as the Ethiopian General Secondary Education Certificate Examination and the Ethiopian Higher Education Entrance Examination, which determine their future educational and career prospects and can cause stress. Adolescent developmental concerns such as identity formation and peer pressure, as well as a lack of mental health resources and support networks in high school, may all lead to higher stress levels (64). The shift from primary to secondary school, which requires acclimating to a new environment and a more rigorous curriculum, can be stressful (65). Furthermore, parental aspirations and peer competition to achieve high grades and gain university entrance may put children under stress. College students, on the other hand, may have more freedom in course choices and control over their academic pathways, which can help to reduce stress. Furthermore, the college environment in Ethiopia may be less competitive and rigorous because students are more focused on basic education than at universities, which have higher expectations for research and internships.

### Factors associated with stress among students in Ethiopia

In this systematic review and meta-analysis, female students were more significantly stressed than male students. This finding is consistent with numerous studies across different countries (66–68). The possible explanation for this is due to a wide difference in the attitude toward the subject, lectures, academic programs, and classroom; female students reported an escalated level of stress than their counterparts (69). Male and female students face various pressures due to social and cultural expectations. Female students frequently suffer increased stress in balancing academic responsibilities with other obligations, such as caregiving and household chores, motivated by cultural expectations about appearance, behavior, and roles within the family and community (70). Moreover, females are more exposed to psychosocial risk factors such as gender-based discrimination, harassment, and violence, leading to chronic stress and anxiety (71). Furthermore, there is a gender difference in coping mechanisms: men who focus on problem-focused coping are better at emotional inhibition than women, who are more focused on emotion-focused coping styles. This makes female students internalize stress and exhibit symptoms of psychological distress, anxiety, and depression more than men (67).

In addition, as evidenced by a number of studies and bolstered by our systematic review and meta-analysis, cigarette smoking is strongly associated with higher stress levels. This finding was in concordance with a study in Iran (72), Malaysia (73), France (74), and the United States (75). This may be due to smokers becoming more socially separated and lonely than non-smokers (76), and the financial and moral guilt effects of tobacco smokers might trigger the development of stress (77). To relieve tension brought on by scholastic demands, social difficulties, and personal problems, a lot of students smoke, but it is ineffective in the long term and can exacerbate stress levels (78). More specifically, this relationship may be explained by the fact that nicotine, the highly addictive component of cigarettes, causes the release of dopamine, which briefly induces feelings of pleasure and relaxation. Nevertheless, once the effects of nicotine wear off, people commonly experience withdrawal symptoms, such as irritation, anxiety, and increased stress (79). Moreover, nicotine contributes to the body’s stress response by changing neurotransmitter pathways in the brain.

Besides, in this study, the odds of developing stress were higher among alcohol drinker students than non-drinkers. This finding is consistent with the previous studies in Uganda (80) and a thematic review among young people in Asian countries (81). In addition, the present result is almost in conformity with WHO fact sheets and highlighted as drinking alcohol is associated with risks of developing mental health and behavioral conditions such as depression, anxiety, and stress (82). It has been observed that university and college students drink alcohol for a variety of reasons, including celebration, decompress, feeling at ease with the other sex, rewarding themselves for working hard, and escaping problems (83). This association can be explained by the fact that drinking alcohol is frequently employed as a coping strategy to lessen stress, but this can create a vicious cycle in which drinking alcohol momentarily lowers tension only for a short time and it returns often more intensely after the effects wear off (84). Frequent alcohol consumption can harm one’s general health, impair judgment, inefficiency in educational achievement, impaired relationships, and interfere with sleep, all of which can lead to more stress (85). Stress levels can be increased by the social and academic fallout from binge drinking.

The results of this systematic review and meta-analysis found that khat chewing is significantly associated with higher levels of stress among students. This finding is consistent with the studies (86, 87). This connection can be due to the stimulant called cathinone in the body of khat chewers that stimulates the secretion of the stress hormone cortisol that makes users more awake but also restless, agitated, and anxious. Frequent use can cause mental health problems like anxiety and mood swings, which increase stress (88). In addition, the protracted nature of purchasing and chewing sessions may interfere with their scholastic obligations and working hours that result in absences from class and poor academic performance. The stimulant effects may cause persistent sleeplessness and sleep loss, which worsen stress (86, 87). Financial strain is another factor, as the cost of Khat can be significant, adding to students’ financial worries and stress. Moreover, the stigma or legal consequences associated with khat use can further contribute to stress through social isolation and legal concerns.

According to the findings of this study, students who live outside of university dorms are more stressed than those who live within. This finding is consistent with research in Lebanon and the United States (68, 89). One possible explanation is that living in a university dormitory provides students with a strong social network and a sense of community, and access to on-campus amenities such as libraries, study rooms, counseling services, and recreational facilities may help reduce stress (68). This may create a favorable environment to participate in extracurricular activities, engage in university life, communicate with peers, and build social networks, all of which are necessary for stress reduction (4). In contrast, students who live off campus may feel alone, separated from the academic environment, have limited access to these networks, and struggle to obtain the resources they require, all of which may contribute to increased stress. Furthermore, commuting owing to the unpredictability of traffic and public transit for off-campus students may diminish time available for studying, resting, and leisure activities that aggravate stress (91).

Moreover, this systematic review and meta-analysis also highlight that students from rural areas experience higher stress levels compared to their urban counterparts. This is in agreement with a study in Saudi Arabia and Syria (92, 93). The possible explanation is that being a rural resident hinders students from the general dearth of modern technology and stable high-speed internet access to online courses, learning resources, and timely completion of their duties. Rural students face financial issues, which add to their stress as they try to pay for living expenses, books, and tuition. Furthermore, transitioning to urban culture while attending school in an urban region can be difficult, with rural students experiencing stress from adjusting to new social standards, as well as loneliness and homesickness (94). However, urban students often have better access to support networks, such as mental health care and academic counseling, whereas rural students may lack these resources. Furthermore, rural students frequently experience mobility issues, such as long commutes or insufficient public transit, which adds to their daily stress by limiting their time for studying, rest, and recreation (95).

Furthermore, this comprehensive systematic review and meta-analysis confirmed that working in an unfavorable environment were factors significantly associated with stress among students. This finding is corroborated with the study done in Ghana and highlighted as a lack of calm and quiet environment could lead to stress for most of the students in higher education (96). This might be students’ perceptions of overcrowding features of school environments, noise and nuisance, inadequate sanitation services, and insecurity may raise their stress level (97). Moreover, this can be explained and resulted in by the inherent characteristics of course workload, lack of leisure time, frequent examinations, and sometimes defective learning materials in academic environments, including high school, college, and university (24).

Finally, in the current systematic review and meta-analysis, we found a significant association between low social support and increased stress levels. This finding is consistent with the study conducted in (98, 99). The possible reason is that the perception of stress is influenced by social support, which buffers against stress and helping individuals cope with challenges. Social assistance provides emotional, academic, financial, and practical support, which can help relieve stress (99). Individuals with strong networks often see stresses as more manageable, whereas those without support may find them overwhelming. Social support from close relationship partners is an important resource for coping with stress (100). In contrast, a lack of social support can result in ineffective coping strategies such as avoidance, which exacerbates stress. Low social support creates feedback loops in which stress causes additional isolation, making people less likely to seek or maintain relationships.

### Methodological considerations

When elucidating the findings of this study, it is crucial to consider a number of methodological concerns. The included studies used various measurement techniques and self-reported questionnaires, which may have under-or over-reported stress levels due to social desirability or recollection bias. In addition, the use of different stress measurement tools and cut-off values to define prevalence presents a challenge in comparing results. To minimize this effect, we performed sensitivity analysis; however, the diversity of tools may add a layer of complexity to interpreting the pooled prevalence. Another important consideration is the heterogeneity observed among the studies. Variation in school levels, geographical setting, and socioeconomic status of students may contribute to variability of stress prevalence. To handle this effect, we apply a random-effects model, and we do subgroups analysis. Furthermore, the reviewed papers with different levels of quality may affect the robustness of our findings. To reduce this, we used the JBI criteria to assess study quality, and we conducted sensitivity analyses to determine the influence of individual studies on the overall results. Finally, confounding variables may have an impact on the validity and interpretation of our findings. To reduce this effect, we performed sensitivity analysis. However, the potential for residual confounding remains, as some factors may not have been fully controlled in all the incorporated studies. Future study should seek to adjust for these factors by collecting additional data and using advanced statistical approaches.

### Strengths and limitations of the study

This study includes a wide range of educational stages, offering a comprehensive picture of stress levels in Ethiopia. The findings have far-reaching significance for mental health professionals, academic institutions, and educational programmers. Furthermore, this study can uncover research gaps, identifying areas that require additional exploration, and its findings can be used to enhance educational policy, mental health programs, and stress management strategies for Ethiopian students. However, the study is not without flaws. The quality of the integrated papers may have an effect on the robustness of the conclusions, as cross-sectional studies can have the potential for indexing bias. While the study is limited to Ethiopia, the findings may not be applicable to other nations or regions with different cultural and educational backgrounds.

## Conclusion

The findings of this systematic review and meta-analysis indicated a high prevalence of stress among students. The study identified female gender, being a rural residence, living outside a university dormitory, khat chewing, having the habit of alcohol consumption, working in an unfavorable environment, poor social support, and cigarette smoking as significant risk factors for stress. To develop coping skills and resilience, integrating mental health education into the curriculum could help students to prevent stress. Furthermore, strategies such as academic support programs, substance use reduction programs, counseling services, and stress management workshops could be beneficial.

## Data Availability

The original contributions presented in the study are included in the article/supplementary material, further inquiries can be directed to the corresponding author.

## Footnotes

^1^ https://www.stata.com
