# Supplementary material for: Prevalence of stress and associated factors among students in Ethiopia: a systematic review and meta-analysis
Source: Front Public Health. 2025 Feb 17;13:1518851. doi: 10.3389/fpubh.2025.1518851 (PMC11874314; doi:10.3389/fpubh.2025.1518851)
Supplement: Supplementary file 1 [file Table_1.pdf]

Supplementary file 1

Table1 shows the searching strategy such keywords, synonyms, data base, searching date and number of articles get.

Key words	Synonym	Data bases, combination, searching date and No of articles found in each database				
		PubMed and its combination	HINARI and its combination	Google scholar and Google	Semantic Scholar	Science direct
		Date		Date	Date	
		August 30/2024	August 30/2024	August 30/2024	August 30/2024	August 30/2024
		No of article = 68	No of article = 46	No of article = 27 and 21	No of article = 15	No of article 10
Prevalence	Magnitude, proportion, epidemiology	**((((((((((((((((((((((((((prevalence [Title/Abstract]) OR (magnitude[Title/Abstract])) OR (proportion[Title/Abstract])) OR (epidemiology[Title/Abstract])) AND (stress*[Title/Abstract])) OR ("psychological stress"[Title/Abstract])) OR ("perceived stress"[Title/Abstract])) OR ("emotional stress"[Title/Abstract])) OR ("mental stress"[Title/Abstract])) OR ("academic stress"[Title/Abstract])) OR ("stress disorders"[Title/Abstract])) AND (student*[Title/Abstract])) OR**	**((Abstract:(prevalence)) OR (Abstract:(magnitude)) OR (Abstract:(proportion)) OR (Abstract:(epidemiology))) AND ((Abstract:(stress*)) OR (Abstract:(psychological stress)) OR (Abstract:(perceived stress)) OR (Abstract:(emotional stress)) OR (Abstract:(mental stress)) OR (Abstract:(academic stress)) OR (Abstract:(stress disorders))) AND ((Abstract:("student*")) OR (Abstract:(adolescent))) AND ((Abstract:(associated factors)) OR (Abstract:(risk factors)) OR (Abstract:(determinates)) OR (Abstract:(correlate***	We apply direct search for Goggle scholar and Google , Web of science and science direct		
Stress	psychological stress, perceived stress, emotional stress, mental stress, academic stress, stress disorders					
Students	high school students, secondary					

	school students, college students, university students, undergraduate students, nursing students, postgraduate students adolescent, young adults	**("high school students"[Title/Abstract])) OR ("secondary school students"[Title/Abstract])) OR ("college students"[Title/Abstract])) OR ("university students"[Title/Abstract])) OR ("undergraduate students"[Title/Abstract])) OR ("nursing students"[Title/Abstract])) OR ("postgraduate students"[Title/Abstract])) OR ("adolescent"[Title/Abstract])) OR ("young adults"[Title/Abstract])) AND ("associated factors"[Title/Abstract])) OR ("risk factors"[Title/Abstract])) OR ("predictors"[Title/Abstract])) OR ("determinants"[Title/Abstract])) OR (correlates[Title/Abstract])) AND (Ethiopia[Title/Abstract])**	**factors)) OR (Abstract:(predictors))) AND (Abstract:(Ethiopia))**	
Associated factors	associated factors, risk factors, predictors, determinants, correlates			
Ethiopia	Ethiopia			
Hint: We use the following filters for PubMed: no observational study, title/abstract, and only English language used.				
